# *HAVCR1* expression might be a novel prognostic factor for gastric cancer

**DOI:** 10.1371/journal.pone.0206423

**Published:** 2018-11-02

**Authors:** Lingling Liu, Zhaoquan Song, Yingchun Zhao, Chao Li, Hua Wei, Ji Ma, Yaowu Du

**Affiliations:** 1 Department of Clinical Laboratory, Shandong Provincial Hospital Affiliated to Shandong University, Jinan, Shandong, China; 2 Clinical Laboratory, Linyi Luozhuang Central Hospital, Linyi, Shandong, China; 3 The 1^st^ Ward of the Department of Paediatrics, Zhangqiu People’s Hospital, Jinan, Shandong, China; 4 Department of NMR, Heilongjiang Provincial Hospital, Harbin, Heilongjiang, China; 5 Department of Endoscopy, Huaihe Hospital, Henan University, Kaifeng, China; 6 Laboratory for Nanomedicine, School of Basic Medical Science, Henan University, Kaifeng, China; University of South Alabama Mitchell Cancer Institute, UNITED STATES

## Abstract

Hepatitis A virus cellular receptor 1 (HAVCR1), which is also known as T-cell immunoglobulin and mucin domain 1 (TIM-1) is a TIM gene family member. In this study, we aimed to characterize the expression profile of *HAVCR1* in GC, its prognostic value and the potential epigenetic mechanism leading to its dysregulation. Bioinformatic analysis was performed by using genomic, clinicopathological and survival data in the human protein atlas (HPA) and the Cancer Genome Atlas (TCGA). Results showed that *HAVCR1* was significantly upregulated at the mRNA and protein level in GC tissues compared to the adjacent normal tissues. In addition, *HAVCR1* upregulation was an independent indicator of shorter OS (HR: 1.698, 95%CI: 1.221–2.361, *p* = 0.002), after adjustment of older age, differentiation status, pathological stages and the presence of residual tumor and was also an independent indicator of shorter RFS (HR: 2.577, 95%CI: 1.583–4.197, *p*<0.001), after adjustment of gender and histological grade. The methylation level of two CpG sites (cg11188031 and cg07320595) was negatively correlated with *HAVCR1* expression. However, only high methylation level of cg07320595 was associated with significantly longer OS (*p* = 0.018) and RFS (*p* = 0.021). Based on these findings, we infer that *HAVCR1* upregulation might serve as a valuable prognostic marker in terms of OS and RFS in GC patients. Cg07320595 might be a critical CpG site influencing *HAVCR1* expression.

## Introduction

Hepatitis A virus cellular receptor 1 (HAVCR1), also known as T-cell immunoglobulin and mucin domain 1 (TIM-1) is a TIM gene family member [1]. It is a class I integral membrane glycoprotein, which contains an N-terminal extracellular immunoglobulin (Ig)-like domain, an extended mucin-like domain, a single transmembrane domain, and a C-terminal short cytoplasmic tail, allowing accessibility to interactions with extracellular proteins [2].

Although *HAVCR1* is expressed in many human tissues, its functional role has not been fully investigated [3]. Previous studies found that the members of this family widely involve in regulating immune cell activity [4, 5]. HAVCR1 is preferentially expressed on Th2 cells, inducing T-cell activation and inhibiting the development of peripheral tolerance [6, 7]. In addition, this molecule is also involved in the moderation of allergic response and asthma [5]. Previous studies found that *HAVCR1* is overexpressed in numerous cancers and its upregulation might be associated with cancer development and progression. In clear cell renal carcinoma (RCC) cells, *HAVCR1* upregulation and its shedding activate the IL-6/STAT-3/HIF-1A axis, which is a signaling pathway enhancing angiogenesis and tumor growth [8]. *HAVCR1* overexpression results in reduced formation and integrity of tight junctions, which have an imperative role in cell to cell adhesion [9]. The disruption of tight junctions is thought to be a cause of enhanced cancer cell dissemination and cancer metastasis [3, 9]. In addition, the cleaved ectodomain of HAVCR1 can be detected in the urine samples from RCC patients, making it a possible biomarker for early detection of RCC [10].

One previous study found that blocking the interaction of TIM-1 and TIM-4 can enhance DC vaccine against gastric cancer (GC) [11]. In this study, using genomic, clinicopathological and survival data in multiple databases, we characterized the expression profile of HAVCR1 in GC, its prognostic value and the potential epigenetic mechanism leading to its dysregulation.

## Patients and methods

### Secondary analysis using data in TCGA-STAD

The level-3 data in TCGA-STAD was downloaded using the UCSC Xena Browser (https://xenabrowser.net/). This database included 415 cases of primary GC tumors and 35 cases of matched normal stomach tissues. These tissues had gene expression quantified by IlluminaHiSeq analysis. No patient had the history of neoadjuvant treatment. Among the patients with RNA-seq data available, 388 cases had intact OS data recorded, while 324 cases had intact RFS data recorded. The genomic, clinicopathological and survival data of the patients, including *HAVCR1* expression (IlluminaHiSeq), age at initial diagnosis, gender, pathological stage, reflux history, histological grade, radiation therapy, targeted molecular therapy, *H*. *pylori* infection, primary therapy outcomes, the presence of residual tumor, living status and recurrence status were downloaded. Primary therapy outcomes were defined as complete remission (CR), partial remission (PR), stable disease (SD), and progressive disease (PD).

The DNA methylation data of *HAVCR1* (measured by Illumina Infinium Human Methylation 450K BeadChip) were also downloaded using the UCSC Xena Browser. Among the GC cases with *HAVCR1* DNA methylation available, 360 GC cases had intact OS data recorded, while 305 cases had intact RFS data recorded.

### Data mining in the Human Protein Atlas

RNA-seq data of *HAVCR1* RNA and protein expression in normal human tissues and human cancer tissues were reviewed via using data generated by the Human Protein Atlas (HPA) (http://www.proteinatlas.org/) [12, 13]. The protein expression was examined by immunohistochemistry and the expression level was scored as not detected, low, medium or high, which is a combination of staining intensity and fraction of stained cells.

### Statistical analysis

Statistical analysis was performed using SPSS 25.0 software package (SPSS Inc., Chicago, IL, USA) or using GraphPad Prism 7.0 (GraphPad Inc., La Jolla, CA, USA). Gene expression between different groups was assessed using one-way ANOVA followed by Turkey’s post-hoc test or Welch’s unequal variances *t*-test. The association between *HAVCR1* expression and the clinicopathological parameters in GC patients was examined by using χ^2^ test by two-sided Fisher’s exact test. Kaplan-Meier survival curves were generated using GraphPad Prism 7.0. The Youden Index of *HAVCR1* RNA expression or its DNA methylation in the Receiver operating characteristic (ROC) analysis for death and recurrence detection were identified and were used as the cutoff in Kaplan-Meier curves. Log-rank test was performed to identify the significance of the difference between the survival curves. Univariate and multivariate Cox regression models were applied to analyze the prognostic significance of *HAVCR1* expression in terms of OS and RFS. Regression analysis was performed to assess the Pearson correlation coefficiency between *HAVCR1* expression and the methylation level of its CpG sites. *p* < 0.05 was considered statistically significant.

## Results

### *HAVCR1* expression was low at both mRNA and protein level in normal stomach tissues

**Fig 1:**
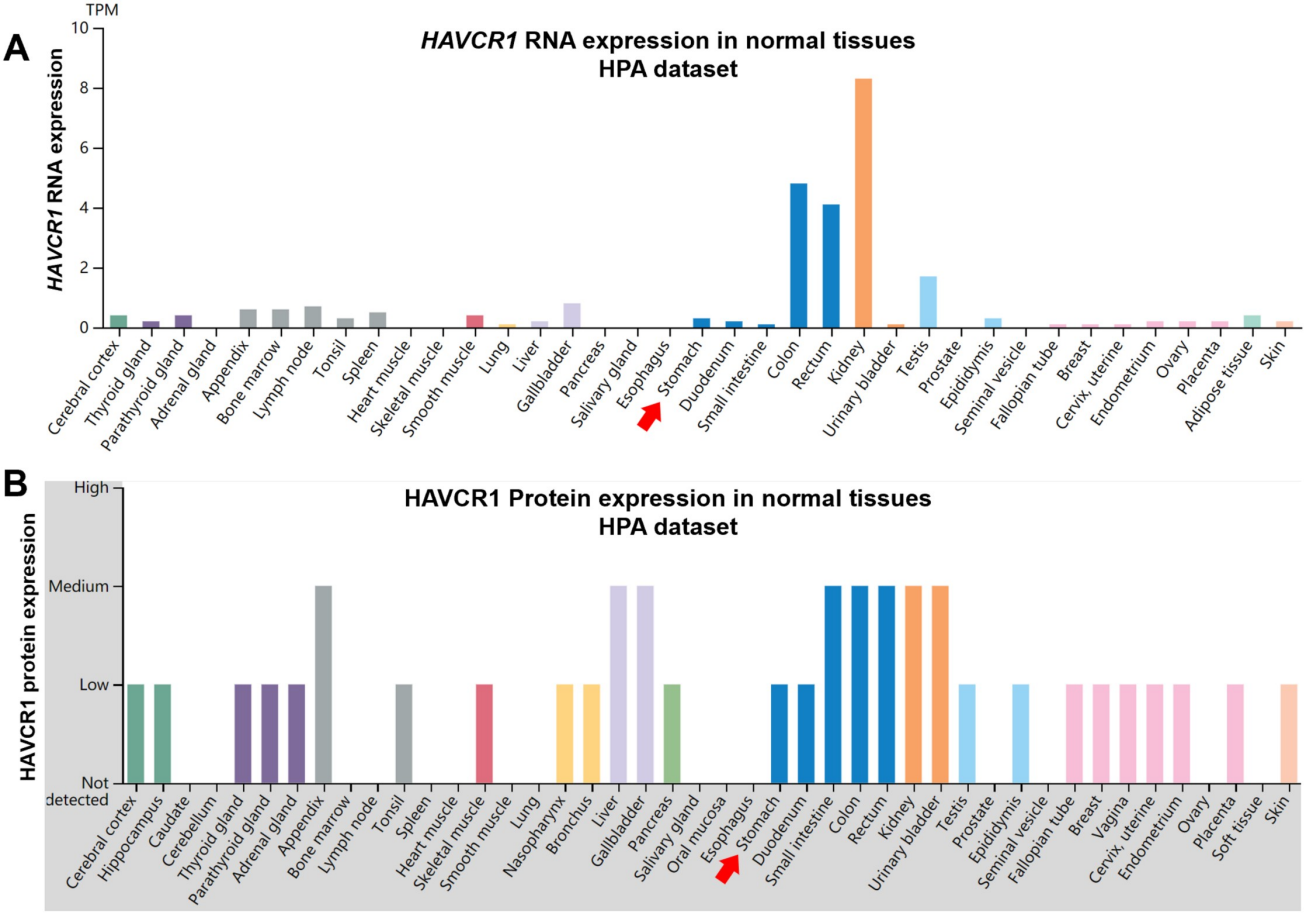
*HAVCR1* expression profiles in normal human tissues. **A**-**B**. The RNA (A) and protein (B) expression profiles of HAVCR1 in normal human tissues. Data credit: Human Protein Atlas. Data summary images were obtained from: v18.proteinatlas.org, via: https://www.proteinatlas.org/ENSG00000113249-HAVCR1/tissue.

Using RNA-seq data and protein expression data (by IHC staining) in the HPA, we characterized *HAVCR1* expression in different normal human tissues. Results showed that *HAVCR1* expression varied significantly in different human tissues (Fig 1A and 1B). The RNA expression was relatively higher in colon, rectum, kidney and testis, compared to other tissues (Fig 1A), while the protein expression was consistent with its RNA expression (Fig 1B). In normal stomach tissues, *HAVCR1* expression was low at both mRNA and protein level (Fig 1A and 1B, red arrows).

### *HAVCR1* expression was upregulated at both mRNA and protein level in GC tissues

**Fig 2:**
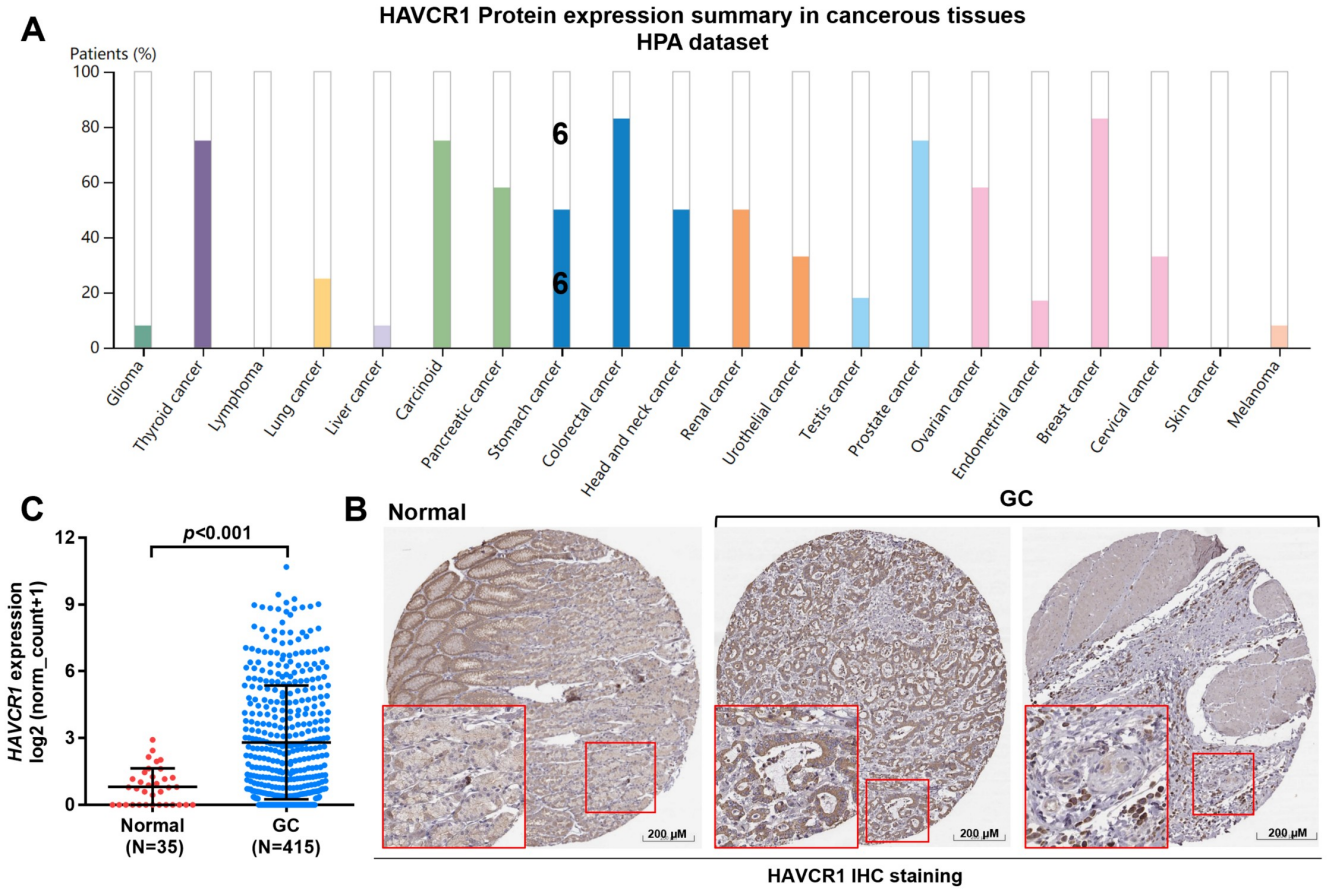
*HAVCR1* expression profiles in human cancer tissues. **A**. The protein expression profiles of HAVCR1 in human cancer tissues. Data credit: Human Protein Atlas. Data summary images were obtained from: v18.proteinatlas.org, via: https://www.proteinatlas.org/ENSG00000113249-HAVCR1/pathology. **B**. Representative IHC images of HAVCR1 expression in normal stomach tissues (left) and in GC tissues (right). Image credit: Human Protein Atlas. Images were obtained from: v18.proteinatlas.org, via: https://www.proteinatlas.org/ENSG00000113249-HAVCR1/pathology/tissue/stomach+cancer#ihc. **C**. Comparison of *HAVCR1* expression between 415 cases of GC tissues and 35 cases of matched normal tissues.

Then, we checked HAVCR1 protein expression in some human tumor tissues (Fig 2A). Among 12 GC tissues examined, 6 cases had medium to high staining (5 medium and 1 high), while the remaining 6 cases had low staining (Fig 2A). Representative IHC image showed that HAVCR1 staining was low in glandular cells in normal stomach tissues (Fig 2B, left). In comparison, the upregulated HAVCR1 was distributed in both cytoplasma and cell membrane in GC tissues (Fig 2B, right). Then, we used RNA-seq data from TCGA-STAD, to examine *HAVCR1* RNA expression between 415 cases of GC tissues and 35 cases of matched normal tissues. Welch’s unequal variances *t*-test confirmed that the GC tissues had significantly upregulated *HAVCR1* expression (Fig 2C).

### Elevated *HAVCR1* expression was associated with poor therapeutic responses and survival outcomes

**Fig 3:**
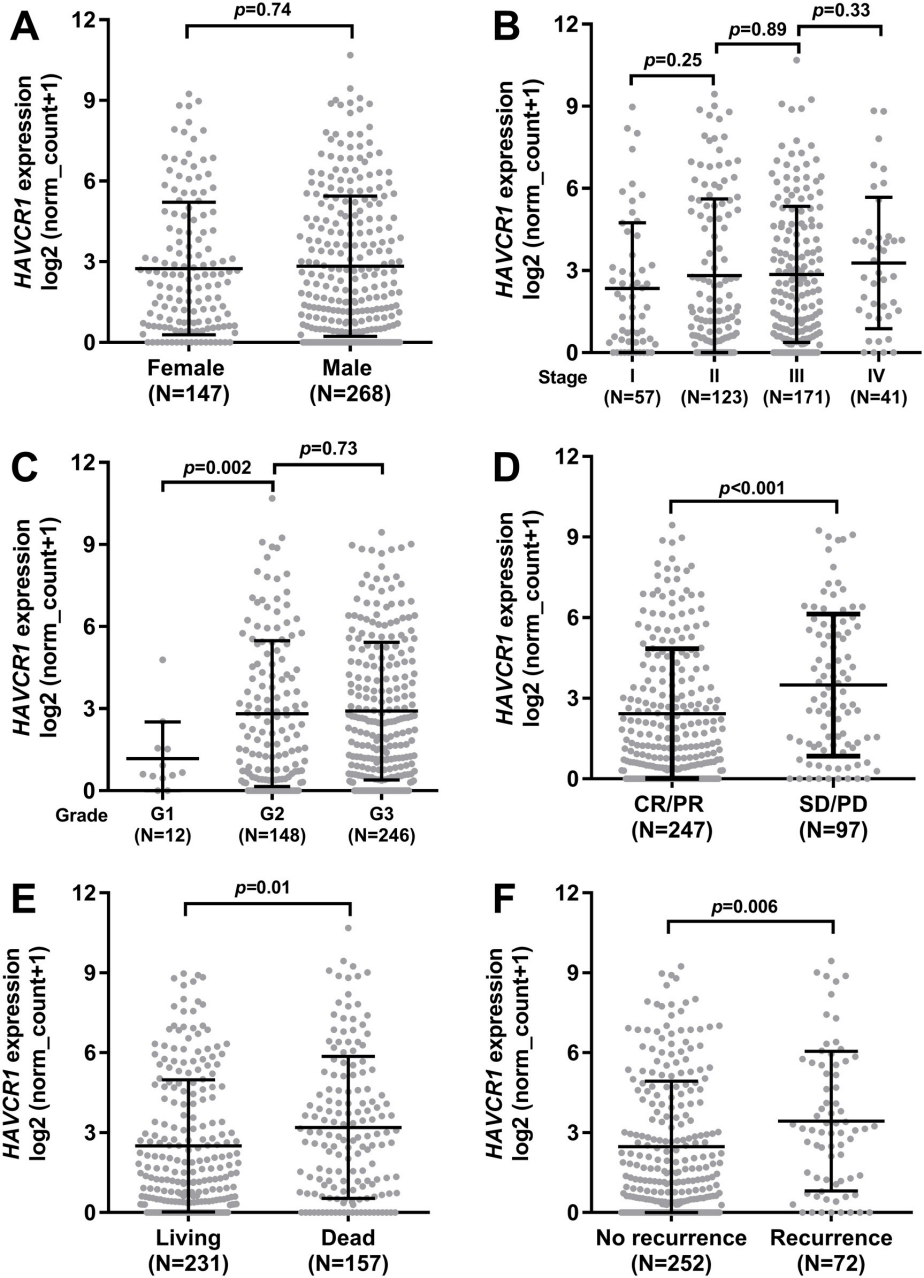
Elevated *HAVCR1* expression was associated with poor therapeutic responses and survival outcomes in GC patients. **A-F**. Comparison of *HAVCR1* RNA expression between female and male patients (A), among patients in different pathological stages (B), different histological grade (C), with different therapeutic responses (D), and with different OS (E) and RFS (F) status.

Then, we checked the difference in *HAVCR1* expression between groups with different clinicopathological parameters and survival outcomes. Results showed that there was no significant difference in *HAVCR1* expression between female and male patients (Fig 3A) or among patients in different pathological stages (Fig 3B). In comparison, the moderately differentiated (G2) tumors had substantially higher *HAVCR1* expression compared to the well-differentiated tumors (G1). No significant difference was observed between G2 and G3 (Poorly differentiated) tumors (Fig 3C). Notably, the patients with responses (CR/PR) to primary therapy had significantly lower (*p*<0.001) *HAVCR1* expression compared to the patients without therapeutic responses (SD/PD) (Fig 3D). Then, we compared *HAVCR1* expression between patients with different survival outcomes. Results showed that the death cases and the cases with recurrence after primary therapy had significantly higher *HAVCR1* expression compared to the control group (Fig 3E and 3F).

### Elevated *HAVCR1* RNA expression was an independent prognostic indicator of unfavorable survival in GC patients

**Fig 4:**
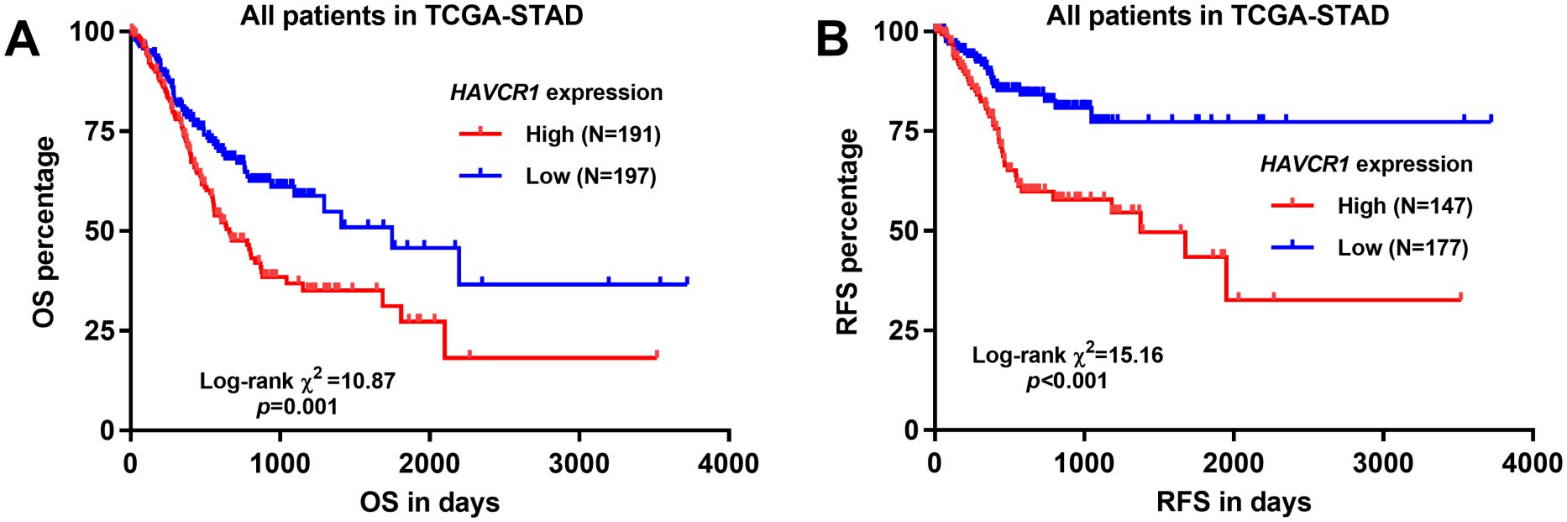
Elevated *HAVCR1* expression was associated with shorter OS and RFS in GC patients. **A-B**. Kaplan-Meier survival curves of OS (A) and RFS (B) in GC patients. The patients were grouped according to the optimal cutoff (Youden Index) of *HAVCR1* expression in ROC analysis for death and recurrence detection.

To assess the prognostic value of *HAVCR1* RNA expression in GC, the patients were divided into two groups according to the optimal cutoff of *HAVCR1* expression. Their clinicopathological parameters and survival outcomes were compared in Table 1 (divided by the optimal cutoff in ROC analysis for death detection). Chi-square analysis showed that the high *HAVCR1* expression group (N = 191) had a significantly higher proportion of patients without responses to primary therapy (*p* = 0.001), deceased cases (*p*<0.001) and with recurrence (*p*<0.001), compared to the low *HAVCR1* expression group (N = 197) (Table 1). By generating Kaplan-Meier survival curves, we found that the high *HAVCR1* expression group had a significantly shorter OS and RFS (Fig 4A and 4B). By performing univariate analysis, we found that older age, poorly differentiated tumor, advanced pathological stages (III/IV), with residual tumor and high *HAVCR1* expression were risk factors of shorter OS (Table 2). Multivariate analysis confirmed that high *HAVCR1* expression was an independent indicator of shorter OS (HR: 1.698, 95%CI: 1.221–2.361, *p* = 0.002), after adjustment of older age, differentiation status, pathological stages and the presence of residual tumor (Table 2). By setting RFS as the survival outcome, we found that male patients, poorly differentiated tumor and high *HAVCR1* expression were risk factors of shorter RFS (Table 3). Multivariate analysis confirmed that high *HAVCR1* expression was an independent indicator of shorter RFS (HR: 2.577, 95%CI: 1.583–4.197, *p*<0.001), after adjustment of other two risk factors (Table 3).

**Table 1:** The characteristics of patients with primary GC.

Parameters		*HAVCR1* expression		*p* value
		High (N = 191)	Low (N = 197)	
**Age (Mean ± SD)**		64.22±10.91	66.35±10.28	0.05
**Gender**	Female	68	68	0.83
	Male	123	129	
**Pathological Stage**	I/II	75	97	0.49
	III/IV	110	93	
	Discrepancy+null	6	7	
**Histological Grade**	G1/G2	67	80	0.25
	G3	121	111	
	GX/null	3	6	
**Reflux history**	No	90	103	0.62
	Yes	22	21	
	null	79	73	
**Radiation therapy**	No	139	155	0.35
	Yes	37	31	
	Discrepancy+null	15	11	
**Targeted molecular therapy**	No	86	106	0.09
	Yes	90	76	
	Discrepancy+null	15	15	
***H*. *pylori* infection**	No	81	73	0.16
	Yes	7	13	
	Null	103	111	
**Primary therapy outcome**	CR+PR	102	139	0.001
	SD+PD	59	35	
	Discrepancy+null	30	23	
**Residual tumor**	R0	154	166	0.13
	R1+R2	19	11	
	RX+null	18	20	
**Recurrence status**	No	106	146	<0.001
	Yes	46	22	
	Null	39	29	
**Living Status**	Living	96	135	<0.001
	Dead	95	62	

Null: data not available; G1: Well differentiated: G2: Moderately differentiated: G3: Poorly differentiated; R0, no residual tumor; R1, microscopic residual tumor; R2, macroscopic residual tumor; RX: The presence of residual tumor cannot be assessed. Patients were separated into two groups according to the Youden Index of *HAVCR1* expression in ROC analysis for OS detection.

**Table 2:** Univariate and multivariate analysis of OS.

Parameters	Univariate analysis				Multivariate analysis			
	*p*	HR	95%CI		*p*	HR	95%CI	
			lower/upper				lower/upper	
**Age** (Continuous)	**0.007**	1.021	1.006	1.037	**<0.001**	1.029	1.013	1.046
**Gender**								
Male		1.000						
Female	0.296	0.835	0.596	1.171				
**Histological grade**								
G1/G2		1.000						
G3	**0.022**	1.479	1.057	2.069	**0.025**	0.668	0.469	0.950
**Pathological stages**								
I/II		1.000						
III/IV	**<0.001**	2.063	1.460	2.916	**<0.001**	1.918	1.333	2.760
**Reflux history**								
Yes		1.000						
No	0.124	1.675	0.868	3.232				
**Residual tumors**								
Yes		1.000						
No	**<0.001**	0.314	0.198	0.498	**<0.001**	0.422	0.260	0.683
**H. pylori infection**								
Yes		1.000						
No	0.193	1.753	0.753	4.082				
***HAVCR1* expression**								
Low (N = 197)		1.000						
High (N = 191)	**0.001**	1.723	1.250	2.374	**0.002**	1.698	1.221	2.361

The cutoff of *HAVCR1* expression was the Youden Index of *HAVCR1* expression in ROC analysis for OS detection.

**Table 3:** Univariate and multivariate analysis of RFS.

Parameters	Univariate analysis				Multivariate analysis			
	*p*	HR	95%CI		*p*	HR	95%CI	
			lower/upper				lower/upper	
**Age** (Continuous)	0.547	0.994	0.973	1.015				
**Gender**								
Male		1.000						
Female	**0.014**	0.504	0.293	0.869	**0.003**	0.437	0.253	0.756
**Histological grade**								
G3		1.000						
G1/G2	**0.014**	0.522	0.311	0.876	**0.010**	0.502	0.298	0.845
**Pathological stages**								
**I/II**		1.000						
**III/IV**	0.803	1.061	0.664	1.696				
**Reflux history**								
Yes		1.000						
No	0.207	1.832	0.716	4.689				
**Residual tumors**								
Yes		1.000						
No	0.150	0.538	0.232	1.252				
**H. pylori infection**								
Yes		1.000						
No	0.334	2.043	0.479	8.710				
***HAVCR1* expression**								
Low (N = 177)		1.000						
High (N = 147)	**<0.001**	2.538	1.561	4.127	**<0.001**	2.577	1.583	4.197

The cutoff of *HAVCR1* expression was the Youden Index of *HAVCR1* expression in ROC analysis for RFS detection.

### Bioinformatic analysis of the epigenetic mechanism underlying *HAVCR1* dysregulation in GC

**Fig 5:**
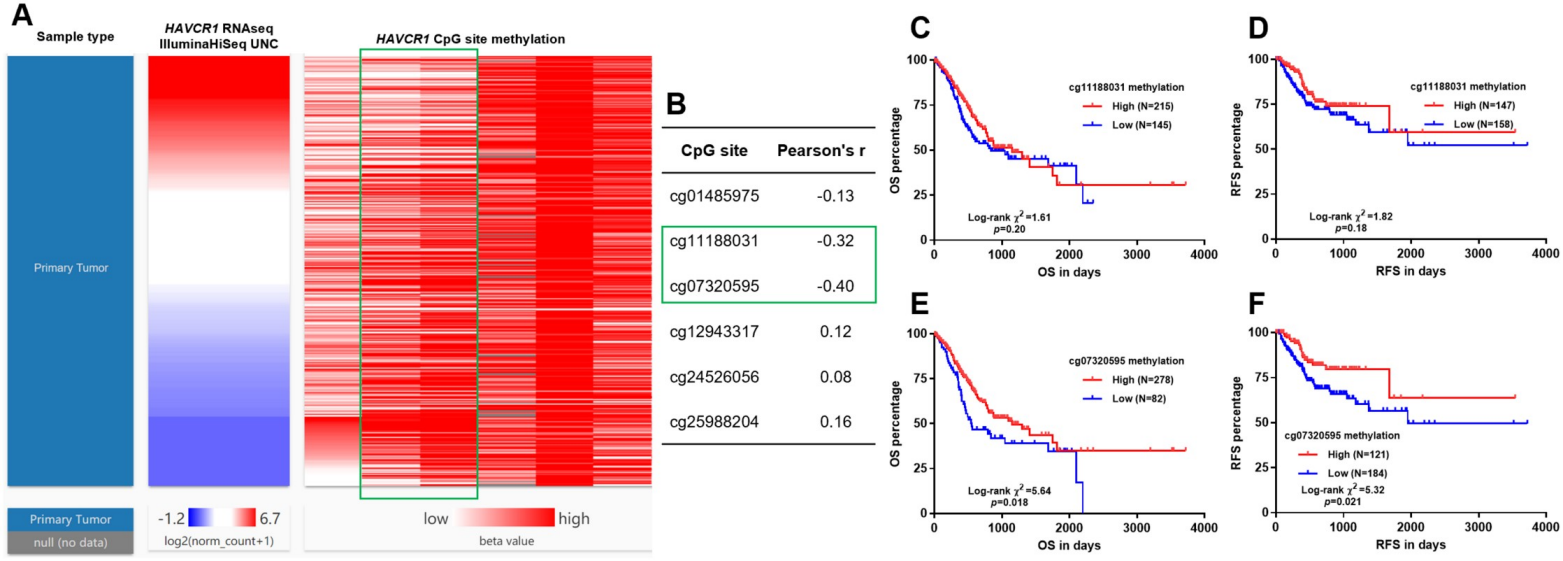
Bioinformatic analysis of the epigenetic mechanism underlying *HAVCR1* dysregulation in GC. **A-B**. Heatmap (A) and the corresponding regression analysis (B) of the correlation between *HAVCR1* expression and the methylation of its CpG sites. **C-F**. Kaplan-Meier survival curves of OS (C and E) and RFS (D and F) in GC patients. The patients were grouped according to the optimal cutoff of cg11188031 (C-D) and cg07320595 (E-F) methylation in ROC analysis for death and recurrence detection respectively.

Using DNA methylation data obtained from Illumina Infinium Human Methylation 450K BeadChip, we examined the methylation status of the CpG sites of *HAVCR1* gene. Heatmap and the corresponding regression analysis found that the methylation level of two CpG sites (cg11188031 and cg07320595) were negatively correlated with *HAVCR1* expression (Pearson’s r = -0.32 and -0.40 respectively) (Fig 5A and 5B). Then, we checked whether the methylation level of these two sites was related to OS and RFS. Kaplan-Meier curves showed that the methylation status of cg11188031 was not related to OS or RFS (Fig 5C and 5D). In comparison, high methylation level of cg07320595 was associated with significantly longer OS (*p* = 0.018) and RFS (*p* = 0.021) (Fig 5E and 5F).

## Discussion

There are three HAVCR family of receptors identified in humans, including HAVCR1, HAVCR2 and HAVCR3. HAVCR2, which is also known as TIM-3, have been widely studied in GC. It is a T helper type 1 (Th1)-specific cell surface molecule that regulates Th1 responses and induces peripheral tolerance [5]. Its expression on NK cells is induced by the pathological development of GC and might act as an independent prognostic factor [14]. Its upregulation on monocyte/macrophages might contribute to GC progression via stimulating monocyte to secrete IL-6, IL-8, and IL-10 [15]. TIM-3 also negatively regulates GC antigen-specific CD8(+) T cell [16]. CD8(+)PD-1(+)TIM-3(+) T cells were more impaired in IFN-gamma, TNF-alpha and IL-2 production compared to the PD-1(+)Tim-3(-) or PD-1(-)Tim-3(-) subsets and were less cytotoxic to GC cells [16]. However, no study examined the expression profile of *HAVCR1* and its functional role in GC.

In this study, using data from the TCGA-STAD, we found that *HAVCR1* was significantly upregulated at the mRNA and protein level in GC tissues compared to the adjacent normal tissues. In addition, we also demonstrated that its upregulation was an independent indicator of shorter OS (HR: 1.698, 95%CI: 1.221–2.361, *p* = 0.002), after adjustment of older age, differentiation status, pathological stages and the presence of residual tumor and was also an independent indicator of shorter RFS (HR: 2.577, 95%CI: 1.583–4.197, *p*<0.001), after adjustment of gender and histological grade. These findings suggest that *HAVCR1* expression might be a novel prognostic factor for gastric cancer. Previous studies HAVCR1 shedding enhances IL-6 expression in RCC, which subsequently activates the STAT-3 pathway, leading to increased HIF-1α expression [8, 17]. In GC, This pathway also mediates a reciprocal crosstalk between cancer-derived mesenchymal stem cells and neutrophils [18], resistance to trastuzumab [19] and epithelial to mesenchymal transition [20], thereby facilitating cancer progression and metastasis. These mechanisms might help to explain the association between aberrantly expressed *HAVCR1* and the poor survival outcomes of GC.

Besides the specific upregulation in several human cancers, the monoclonal antibody binds to HAVCR1 can be internalized into the cells, making it an attractive target for antibody-mediated therapy, such as antibody-drug conjugates (ADCs) in certain cancers [21–23]. In this study, we observed that *HAVCR1* upregulation was associated with poor responses to primary therapy. In the future, it is meaningful to further explore the potential of HAVCR1 as a target of ADCs in GC.

To explore the potential mechanism underlying its dysregulation, we examined the correlation between *HAVCR1* RNA expression and the methylation level of its CpG sites. Results found two CpG sites (cg11188031 and cg07320595) that were negatively correlated with *HAVCR1* expression. However, by performing survival analysis, only high methylation level of cg07320595 was associated with significantly longer OS (*p* = 0.018) and RFS (*p* = 0.021). Based on these findings, we infer that cg07320595 might be a critical CpG site influencing *HAVCR1* expression.

## Conclusion

*HAVCR1* upregulation might serve as a valuable prognostic marker in terms of OS and RFS in GC patients. Cg07320595 might be a critical CpG site influencing *HAVCR1* expression.
